# Hydrogen sulphate-based ionic liquid-assisted electro-polymerization of PEDOT catalyst material for high-efficiency photoelectrochemical solar cells

**DOI:** 10.1038/s41598-017-11916-4

**Published:** 2017-09-15

**Authors:** Buket Bezgin Carbas, Mahir Gulen, Merve Celik Tolu, Savas Sonmezoglu

**Affiliations:** 1 Department of Energy Systems Engineering, Karamanoglu Mehmetbey University, Karaman, Turkey; 2 Conductive Polymers and Energy Applications Laboratory, Karamanoglu Mehmetbey University, Karaman, Turkey; 3 Nanotechnology R&D Laboratory, Karamanoglu Mehmetbey University, Karaman, Turkey; 4 Department of Metallurgical and Materials Engineering, Karamanoglu Mehmetbey University, Karaman, Turkey

## Abstract

This work reports the facile, one-step electro-polymerization synthesis of poly (3,4-ethylenedioxythiophene) (PEDOT) using a 1-ethyl-3-methylimidazolium hydrogen sulphate (EMIMHSO_4_) ionic liquid (IL) and, for the first time its utilization as a counter electrode (CE) in dye-sensitized solar cells (DSSCs). Using the IL doped PEDOT as CE, we effectively improve the solar cell efficiency to as high as 8.52%, the highest efficiency reported in 150 mC/cm^2^ charge capacity, an improvement of ~52% over the control device using the bare PEDOT CE (5.63%). Besides exhibiting good electrocatalytic stability, the highest efficiency reported for the PEDOT CE-based DSSCs using hydrogen sulphate [HSO_4_]^−^ anion based ILs is also higher than platinum-(Pt)-based reference cells (7.87%). This outstanding performance is attributed to the enhanced charge mobility, reduced contact resistance, improved catalytic stability, smoother surface and well-adhesion. Our experimental analyses reveal that the [HSO_4_]^−^ anion group of the IL bonds to the PEDOT, leading to higher electron mobility to balance the charge transport at the cathode, a better adhesion for high quality growth PEDOT CE on the substrates and superior catalytic stability. Consequently, the EMIMHSO_4_-doped PEDOT can successfully act as an excellent alternative green catalyst material, replacing expensive Pt catalysts, to improve performance of DSSCs.

## Introduction

Dye-sensitized solar cells (DSSCs) have emerged as a promising alternative to traditional silicon solar cells owing to their acceptable energy conversion efficiency, low cost/performance ratio, environmental friendliness and simple production process^1–5^. A typical DSSC is fabricated on a titanium dioxide (TiO_2_) photo-anode sensitized with a ruthenium/organic dye as the light-harvesting media and has a redox electrolyte consisting of ${I}_{3}^{-}/{I}^{-}$ and a platinum (Pt) counter electrode (CE). The CE collects the electrons flowing from the external circuit and catalyzes the reduction of ${I}_{3}^{-}$ to *I* ^−^ by pumping the collected electrons into the electrolyte solution^6^. As a CE material, Pt is a superb catalyst due to its good electronic conductivity and electrocatalytic activity^7, 8^. DSSCs assembled with a Pt CE have been shown to achieve power conversion efficiency (PCE) of more than 12%^9^. However, the high price, low availability and corrosion potential of Pt in the triiodide solution are serious drawbacks to the cost-effective fabrication and long-term stability of DSSCs. Consequently, it is imperative to develop alternative CE materials with relatively high catalytic activity, conductivity and chemical stability. Hereby, some alternative materials have been explored, such as inorganic semiconductors, carbonaceous materials and conducting polymers (CPs)^10–16^. CPs such as polyaniline (PANI), polypyrrole (PPy) and polythiophene (PTh) have been commonly employed as CE materials owing to their good conductivity, simple polymerization process with good homogeneity, large surface area, high chemical stability and high electrocatalytic activity^17–20^. Among CPs, poly (3,4-ethylenedioxythiophene) (PEDOT), a derivative of PTh, is the most prominent polymer because it has higher conductivity (300–500 S cm^−1^) than PANI (0.1–5 S cm^−1^) or PPy (10–50 S cm^−1^), higher catalytic activity for the reduction of ${I}_{3}^{-}$ to *I* ^−^ and excellent environmental stability in air and electrolyte media^21, 22^.

Various methods have been used to prepare PEDOT conducting polymer as a CE in DSSCs, including chemical polymerization, electro-polymerization, simple *in situ* polymerization and thermal polymerization. Of these techniques, the electro-polymerization technique is the simplest, most reproducible and cost-effective route to fabricate a highly conductive and high quality PEDOT film. Furthermore, using this method, a film can be directly deposited on the surface of the conducting substrate, and it allows the polymer to be deposited only on the desired areas. It also allows easy control of film thickness by varying deposition charge. The electro-polymerization process typically is performed in polaric organic media or in the presence of water. With electro-polymerization, the growing medium, that is, the electrolyte has a critical impact on the electrical, structural, morphological and especially electrocatalytic properties of the polymers. The use of a water-based electrolyte results in the formation of a non-conducting or passive polymer film. However, the high vapor pressure of organic electrolytes makes evaporation difficult to control during polymer formation/deposition, which can lead to unknown changes in the microstructure of the resultant polymer^23^. The use of room temperature ionic liquids (RTILs) as solvents or additives allows the electrocatalytic, electronic and morphologic properties of the pertinent polymer to be controlled and enhanced because of the negligible vapor pressure, functional ionic groups, wide electrochemical window, high electrochemical stability and attractive hydrophobicity of RTILs^24–27^. It is well known that a hydrophobic medium facilitates deposition of uniform, well-adhered, thick films, whereas highly polar media, which interact weakly with EDOT, are known to cause polymer to flake off the substrate following growth^28^. Furthermore, in contrast to the doping of PEDOT in traditional organic electrolytes alone, wherein only the anions are integrated in the polymer structure, when PEDOT is electro-polymerized in RTILs, both the cation and the anion of the RTIL form an integral part of the polymer matrix, and thus the conductivity of the polymer might be improved^29^. For example, Senadeera *et al*. proposed solid-state polymer-sensitized solar cells with various RTIL-doped PEDOT films as the hole-transporting active media. Imidazolium salts, such as EMIMTf_2_N and LiTf_2_N (see SI), were employed to enhance the conductivity and charge transferability of π-conjugated CPs^30^. Imidazolium cations can be incorporated into a polymer matrix and perform crucial roles in producing a highly porous structure for the CP. This is desirable because the catalytic activity of the polymer is almost completely dictated by its large active surface area induced by nanometer-scaled grain size. In another study, Ahmad *et al*. used BMITFSI, BMPyTFSI and EMIFAP (please see SI) as electrolytes for the electro-polymerization of poly(3,4-propylenedioxythiophene) (PProDOT), another derivative of PTh. The DSSCs assembled with BMITFSI-, BMPyTFSI- and EMIFAP-doped PProDOT CEs achieved very high conversion efficiencies of 9.12%, 9.25% and 9.12%, respectively, comparable to that observed with the corresponding DSSC using a Pt CE (9.53%)^28^. More recently, Li *et al*. investigated the impacts of cationic and anionic dopants on the photovoltaic performances of DSSCs equipped with EMIBF_4_^−^, HMIBF_4_^−^, DMIBF_4_^−^, HMIPF_6_^−^, HMISO_3_CF_3_^−^ and HMITFSI^−^ (see SI) doped PEDOT CEs. All of the DSSCs with the IL-doped PEDOT CEs exhibited higher PCEs than the cell with bare PEDOT due to their larger active surface areas, higher conductivities and attractive hydrophobicities. Moreover, the DSSC with HMITFSI-doped PEDOT CE demonstrated a higher PCE (8.87%) than the Pt CE-based cell (8.09%)^27^.

Taking into account above-mentioned short literature survey, the number of ILs composed of different cation and anion combinations has been performed as solvent or additive in the electro-polymerization of CPs. The physical (hydrophobicity and morphology) and chemical (reaction pathway and stability) properties for polymers change depending on the chosen ions in the IL structure^27^. The popularity of 1,3 dialkyl imidazolium cation based IL usage during electro-polymerization of PEDOT is indisputable due to the high conductive and electrochemically stable polymer formations. Most commonly employed best performance IL anions for PEDOT electrode formation have been fluorous anions, such as PF_6_^−^, BF_4_^−^, CF_3_SO_3_^−^ and (CF_3_SO_3_)_2_N^−^ as a CE for DSSCs^27^. Despite their widespread usage in corresponding applications, their safety and cost are big concerns^31^. For that reason, it is compulsive to introduce new non-fluorous anion based ILs into polymer structure for usage in DSSC applications as a CE. It may be a feasible approach for high performance and environmental friendly DSSCs. Surprisingly, hydrogen sulphate [HSO_4_]^−^ anion based ILs doped PEDOT CPs has never been studied in DSSCs as CE. For this purpose, we chose the 1-ethyl-3-methylimidazolium hydrogen sulphate (EMIMHSO_4_) IL because of its large electrical conductivity, high charge mobility and superior catalytic activity^32, 33^. Furthermore, as this hydrogen sulphate [HSO_4_]^−^ anion based IL can be employed using simple solution-processed at room temperature, this approach is compatible with the large-scale manufacturing against the limitation of the large-scale application of DSSCs resulting from high cost of Pt.

Herein, PEDOT CPs by incorporating EMIMHSO_4_ ILs as an additive in the electrolyte during electro-polymerization were employed for the first time as CE to develop a DSSC with high stability and efficiency. In order to determine the influence of EMIMHSO_4_ and the polymerization charge capacity on the electrochemical stability and photovoltaic performances of the DSSCs, the electro-polymerization of PEDOT was performed in the range of 50–300 mC/cm^2^ with increments of 50 mC/cm^2^, using EMIMHSO_4_ IL as additive. EMIMHSO_4_ IL-doped PEDOT CE electro-polymerized at 150 mC/cm^2^ charge capacity reached the highest efficiency up to 8.52% among the IL-assisted PEDOT CEs. Moreover, this efficiency is higher than Pt-based CE (7.87%) due to its excellent electrocatalytic activity/stability, better charge transfer kinetics and larger active surface area. These results present that EMIMHSO_4_ IL not only acted as a good conducting binder for PEDOT conducting polymer, but also provided a continuous polymer matrix to increase the electron transfer pathways and stability. In shortly, EMIMHSO_4_ IL-assisted PEDOT CE is a promising CE alternative to expensive Pt CEs for use in high efficiency Pt-free DSSCs.

## Results and Discussion

### Part A: Impact of the EMIMHSO_4_ IL on structural, electrochemical and photovoltaic properties of PEDOT CE

**Figure 1:**
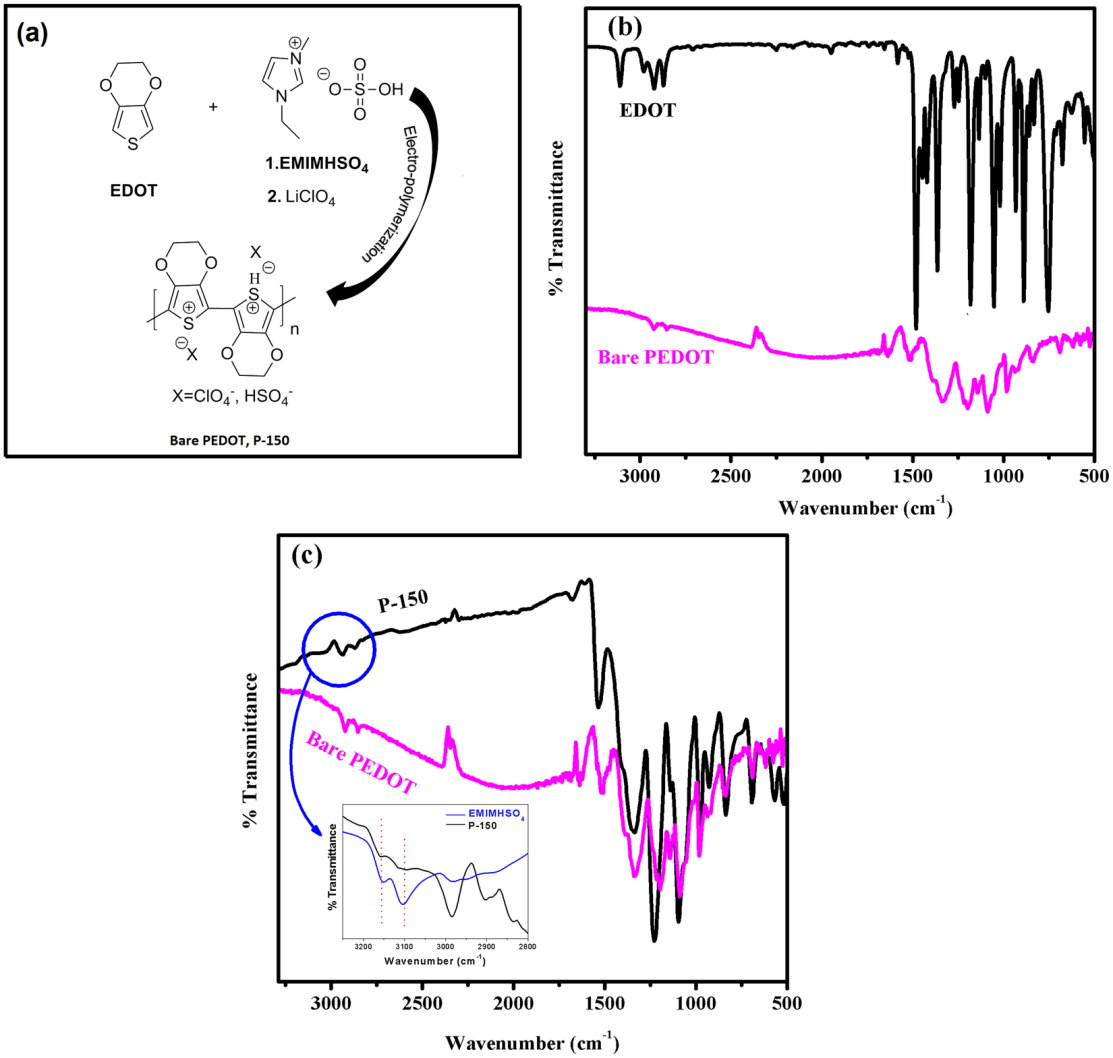
(**a)** Scheme of electro-polymerization process for PEDOT CEs (**b)** FTIR spectra of EDOT and bare PEDOT film. (**c)** FTIR spectra of both bare PEDOT and P-150 films (Inset: the FTIR spectrum comparison between EMIMHSO_4_ and P-150).

A summary of FTIR spectroscopy results for EDOT monomer, its polymer films, bare PEDOT, EMIMHSO_4_ and P-150 based on the literature^34–37^ is shown in Table S1. Firstly, the polymerization of EDOT monomer from α, α’ positions was proven. Most of the bands observed in the FTIR spectrum of the monomer are also present in that of bare PEDOT and P-150. The peak at 3120 cm^−1^, due to C–H stretching (-hydrogen) of the external EDOT units, disappears, whereas other peaks remain, indicating that polymerization proceeds via the C-2 and C-5 position of external EDOT units. The peak at approximately 1090 cm^−1^ is due to the ClO^4−^ dopant (Fig. 1b). The effect of EMIMHSO_4_ on the PEDOT phase structure could also be due to an interaction between the polymer and IL. Thus, the FTIR spectra of the P-150 films were compared with bare PEDOT and EMIMHSO_4_ as references (Fig. 1c). When the FTIR spectra of P-150 and bare PEDOT are compared with the spectra of the individual components, almost all of the bands in the P-150 spectrum can be accounted for. When the spectra of bare PEDOT and P-150 are compared, extra peaks are noted at approximately 3150 and 3105 cm^−1^ in the P-150 film due to the imidazolium cation in EMIMHSO_4_ (see the inset of Fig. 1c). The fingerprint region of pure EMIMHSO_4_ shows the bands attributable to HSO_4_^−^ vibration mode at 1220 cm^−1^ assigned to –O-S stretching; these are also expected in the spectrum of P-150. The strong peak at 836 cm^−1^ together with the weak peak at 760 cm^−1^ also correspond to S-OH stretching. In the P-150, some peaks overlapped with the bare PEDOT vibration modes and shifted to higher wavenumbers, as shown in Table S1. Taken together, these results demonstrate that IL EMIMHSO_4_ is incorporated into the polymer matrix P-150 film as an additive.

**Figure 2:**
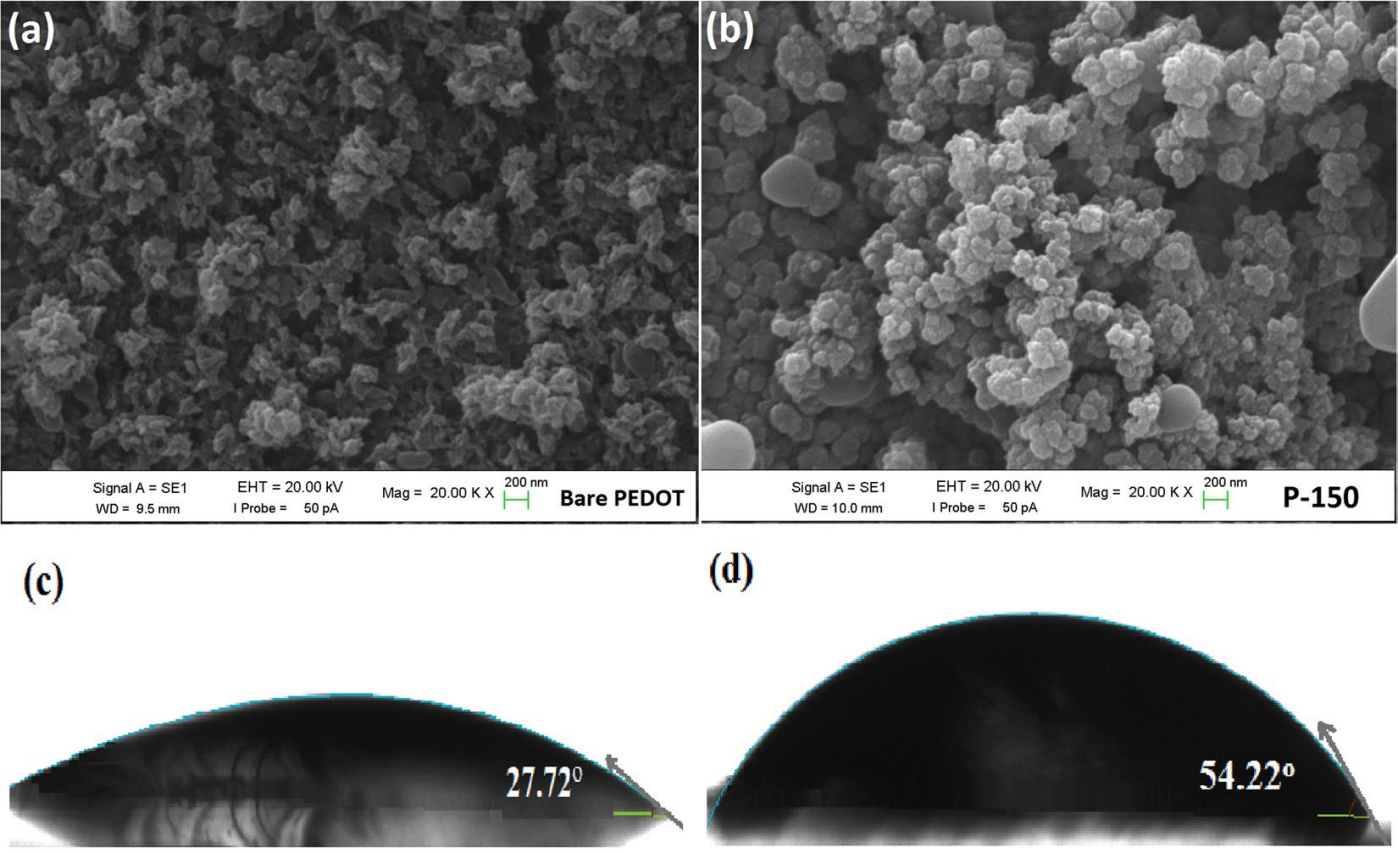
SEM micrographs of the (**a)** bare PEDOT and (**b)** P-150 CEs, contact angle of (**c)** bare PEDOT and **d)** P-150 CEs.

SEM analyses were performed to understand the influence of IL on surface morphology and the growth mechanism of PEDOT CE. Figure 2a and b displays SEM surface morphologies of the different CEs, including PEDOT CE electro-polymerized using EMIMHSO_4_ IL as additive and without IL at 150 mC/cm^2^ polymerization charge capacity. The SEM images shown in Fig. 2a and b clearly demonstrate that a drastic morphological modification occurs on the surface of bare PEDOT CE when EMIMHSO_4_ IL is incorporated into the polymerization medium, owing to the insertion of both cation and anion in the polymer matrix^22^. Throughout the electro-polymerization process, ions are incorporated into the polymer matrix as dopants, and this dominates the growth pattern, resulting in a narrow distribution confined to nanometer-sized domains because of the increased viscosity of electro-polymerization bath and diffusion limitation^38^. Moreover, as the bare PEDOT CE shows a smooth surface morphology with randomly distributed particles, the P-150 CE exhibits a densely packed structural morphology that is comprised of uniform globular grains of approximately 80–150 nm in size. Thus, the conductivity of the polymer may be improved due to more interconnected grains (dense packing) with uniform distribution of globular grains. Furthermore, due to the incorporation of the IL, the P-150 CE exhibited a more porous structure and larger active surface area than bare PEDOT CE. The highly porous morphology and larger surface area offer two advantages: i) a short diffusion path for ions, resulting in a fast charge transport, and ii) improved diffusion of iodide/triiodide redox species due to the increase in catalytic active sites for trapping redox liquid electrolyte in the CE^39^. Furthermore, the large surface area arising from the porous nature of the P-150 CE is advantageous for DSSC performance because it can increase the short circuit current density (*J*_sc_)^40^. To further investigate the surface properties of bare PEDOT and P-150 CEs, AFM measurements were also conducted and illustrated in Fig. S1. The surface morphologies of CEs captured by AFM show similar trends with SEM results. As we examine the surface roughness that directly affects photovoltaic performance, the P-150 CE film exhibited larger surface roughness (180 nm) compared to bare CE film (160 nm), implying that the P-150 film possesses larger surface area and smaller voltage drop on substrate surface^41, 42^. To understand the effect of hydrogen sulphate based IL on wettability of the surface of the films, the shape of water droplets on surface of the bare PEDOT and P-150 CE have also been monitored by the contact angle measurement as depicted in the Fig. 2c and d, respectively. By incorporating hydrogen sulphate based IL in media, the contact angle abruptly increased from ~28° for bare to ~54° for P-150. This increment indicates that the structure convert hydrophilic to hydrophobic with integration of anion (HSO_4_^−^) based IL due to an accompanying increase in the surface roughness and pores on the sample surface, thus leading to improved cell efficiency^43^.

**Figure 3:**
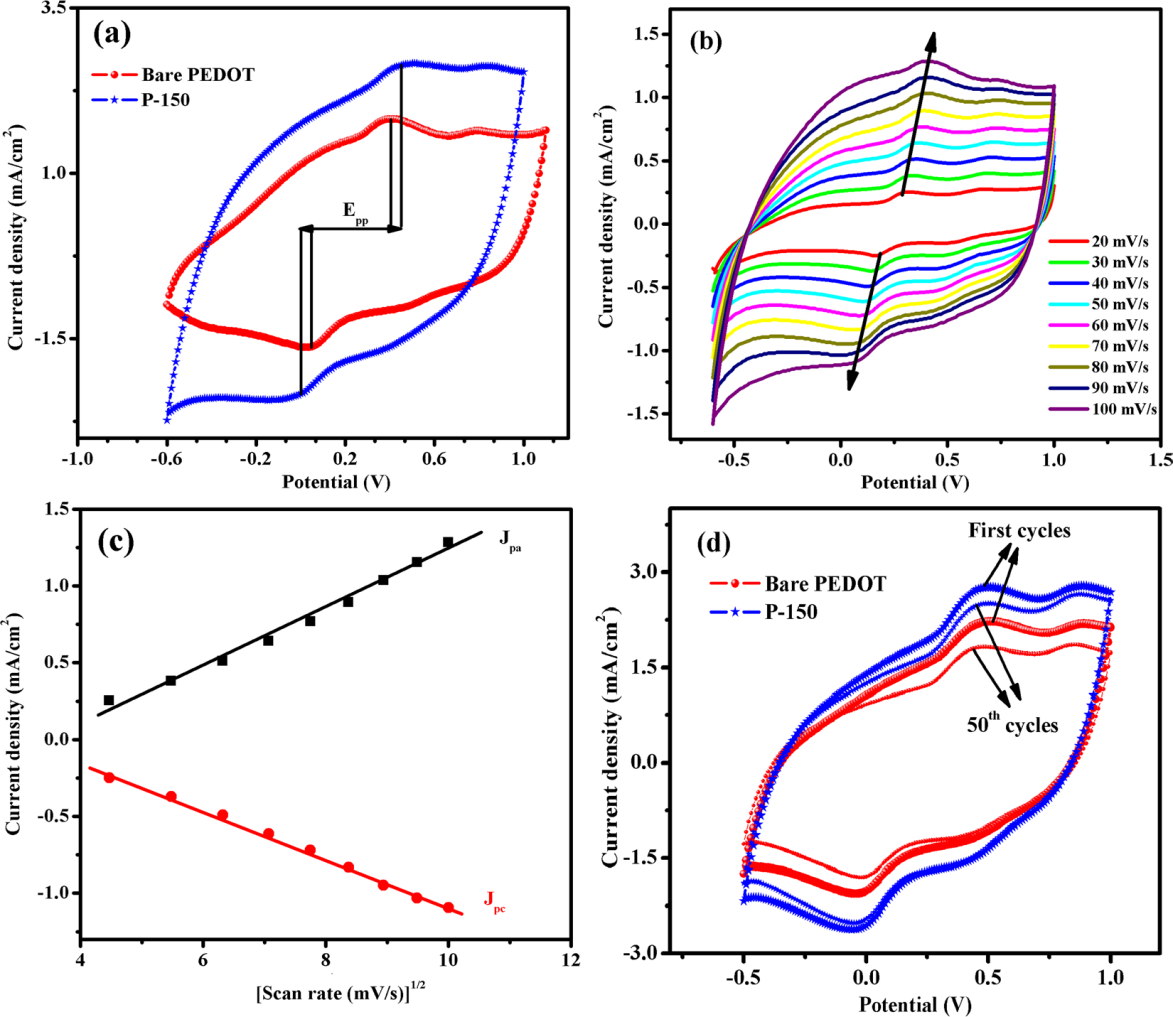
(**a)** CV plot of P-150 and bare PEDOT CEs obtained at 100 mVs^−1^ scan rate, (**b)** CVs for P-150 CE with various scan rates (from inner to outer: 20, 30, 40, 50, 60, 70, 80, 90 and 100 mVs^−1^) and (**c)** the relationship of *J*_*pa*_ and *J*_*pc*_ as a function of the square root of the scan rate for P-150 CE. (**d)** 50 consecutive voltammetry cycles for bare PEDOT and P-150 CEs in the medium of 0.1 M LiClO_4_/ACN with ${I}_{3}^{-}/{I}^{-}$ electrolyte at a scan rate of 100 mVs^-1^.

As depicted in Fig. 3a, to elucidate the electrocatalytic activity of the P-150 and bare PEDOT CEs toward the reduction of triiodide in the redox shuttle of DSSCs, CV measurements were conducted over the range of −0.6 to 1.0 V. The peak current density (*J*_*pd*_) and peak separation between the anodic and cathodic peaks (*E*_*pp*_), which is negatively correlated with the standard electrochemical rate constant of the redox reaction, are useful criteria for evaluating the overall electrocatalytic activity of a CE^6^. According to the CV curves shown in Fig. 3a, the *J*_*pd*_ value for the P-150 CE that was electro-polymerized using EMIMHSO_4_ IL as additive is 2.64 mA/cm^2^, compared with 1.82 mA/cm^2^ observed for the bare PEDOT CE. This increase in *J*_*pd*_ response indicates an excellent electrocatalytic ability to reduce triiodide in the electrolyte. The higher conductive P150 CE polymer containing ionic liquid electrolyte with its superior anion/cation association property inside the polymer matrix may also provide a higher *J*_*pd*_ value than bare PEDOT CE^27^. Moreover, the *E*_*pp*_ parameter, which is inversely proportional to the electrocatalytic activity, is slightly higher for the P-150 CE than for bare PEDOT CE. Although *E*_*pp*_ value led to an overpotential loss in the DSSC, conductivity and active surface area of a CE can effect the *J*_*sc*_, fill factor (*FF)* and so the open circuit voltage (*V*_*oc*_) predominantly than the slightly higher *E*_*pp*_ value^44, 45^. Therefore, overall consideration of *J*_*pd*_ and *E*_*pp*_ indicate that P-150 CE possess superior electrocatalytic activity and a much faster rate for the triiodide reduction reaction in comparison with that of bare PEDOT CE. Furthermore, the reversibility of the redox reaction toward ${I}_{3}^{-}/{I}^{-}$ reaction is also a vital parameter for the electrocatalytic activity of a CE, which can be measured from the ratio of *J*_*pa*_ to |*J*_*pc*_|^46^. The ratio of J_pa_/|J_pc_| were determined as ~1.15 and ~1.10 for bare and P-150 CEs, respectively, indicating P-150 CE has a better reversibility for reaction of ${I}_{3}^{-}/{I}^{-}$ in the corresponding DSSC^47^. The electrocatalytic activity increase observed for the P-150 CE is due to the enlarged surface area that results from the porous morphology shown in Fig. 2a and the improved conductivity of the polymer film^48^. As presented in Fig. 3b,c, the relationship between *J*_*pd*_ and the scan rate for P-150 CE were also investigated. The CV curves show that the P-150 CE has an increased scan rate, and the anodic peak current density (*J*_*pa*_) and cathodic peak current density (*J*_*pc*_) are shifted to the positive and negative directions, respectively. As shown in Fig. 3c, the plots of *J*_*pa*_ and *J*_*pc*_ versus the square root of scan rate are almost linear. According to the Langmuir isotherms principle, this linearity shows the diffusion limitation of the redox reactions on the P-150 CE. Furthermore, this linear relationship reveals that the adsorption of iodide species is affected by the redox reaction on the surface of P-150 CE, and furthermore, there is no specific interaction between the ${I}_{3}^{-}/{I}^{-}$ redox couple and P-150 CE^49, 50^. Moreover, the electrochemical stability of bare PEDOT and P-150 CEs were examined via CV method by taking repeated 50 CV cycles for each of polymer film in the medium of 0.1 M LiClO_4_/ACN with ${I}_{3}^{-}/{I}^{-}$ redox electrolyte. The successive CV cycles gave clues about the occurrence of the corrosion or dissolution of these several CE materials. As shown in Fig. 3d, both CEs effectively catalyze the reduction of ${I}_{3}^{-}$ to ${I}^{-}$ many times but P-150 CE has better electrochemical stability and prolonged coexistence in the two types of redox species. While the displayed cyclic electrode retention of P-150 CE has a stability of 94% after 50 cycles, bare PEDOT CE lost 13% of its electrocatalytic activity.

**Figure 4:**
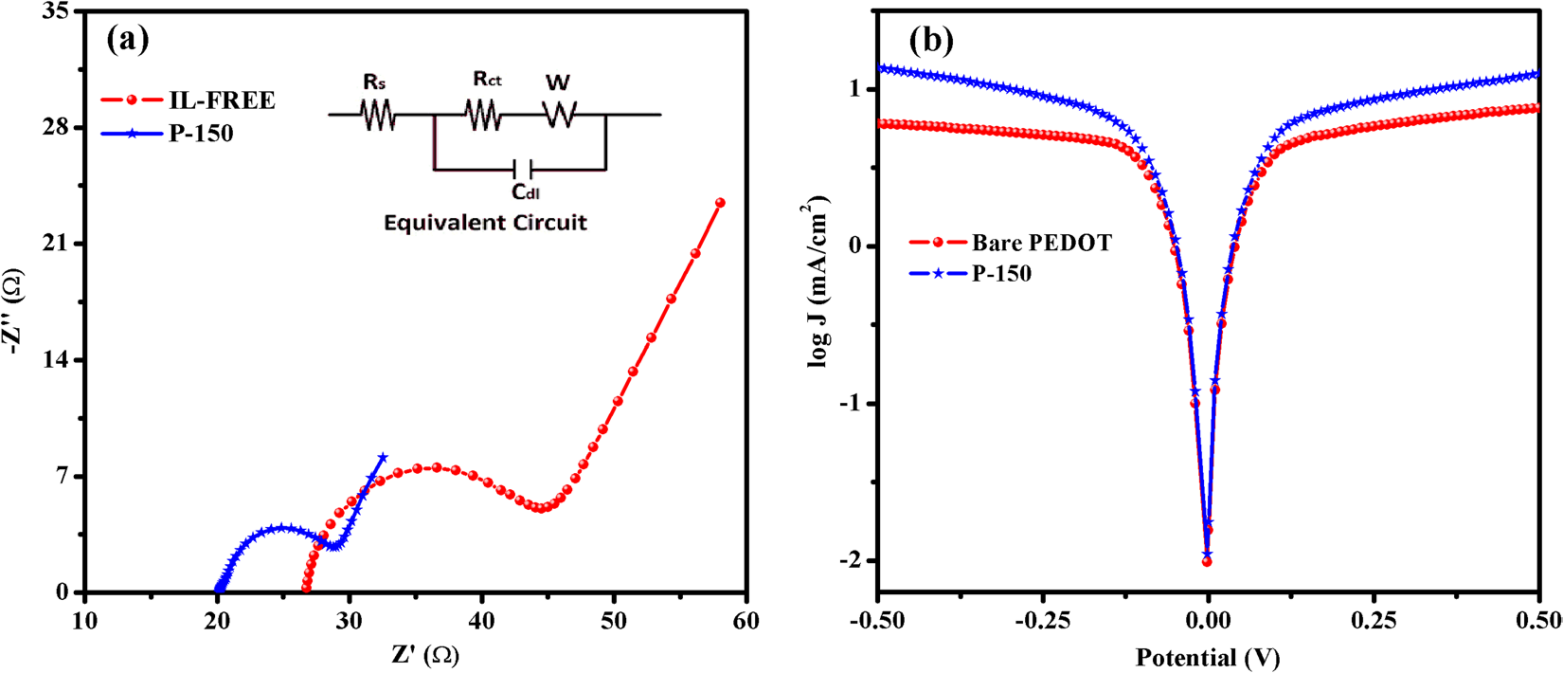
(**a)** Nyquist plot and equivalent circuit (inset) and (**b)** Tafel polarizations of dummy cells using P-150 and bare PEDOT CEs.

To investigate impact of hydrogen sulfate-based IL on charge transfer kinetics and conductivity of PEDOT films, the EIS measurements were carried out in the medium of 0.1 M LiClO_4_/ACN. The impedance plots at open circuit potentials for bare PEDOT and P-150 polymer films are shown in Fig. S2. EIS spectra for both materials have small arcs in high frequency and straight lines in the low frequency regions. At high frequency region, the intercept with x-axis (Z’ axis) gives information about the intrinsic ohmic resistance of internal resistance or equivalent series resistances of electrode and electrolyte solution^51^. While bare PEDOT film has a resistance of 57.6 Ω, the P-150 CE has a lower one (39.2 Ω) in the high frequency region, indicating that the presence of IL for P-150 film could facilitate electron transfer and effectively improved by the doping of IL. In case of low frequency region, a steeper gradient of P-150 shows faster ion diffusion^52^, which will foresee the good electrical conductivity and more importantly the obtaining better performance as a CE in DSSC than bare PEDOT film has. To further investigate the intrinsic interfacial charge transfer and charge transport kinetics at the electrolyte/CE interface, EIS measurements were also performed using electrochemical dummy (symmetric) cells with identical CEs. Figure 4a shows the Nyquist plots for the corresponding equivalent circuit (inset in Fig. 4a) of the symmetric cells prepared with bare PEDOT or P-150 CEs. By fitting the Nyquist plot with the equivalent circuit, the ohmic series resistance (*R*_*s*_), charge transport resistance (*R*_*ct*_) and corresponding double layer capacitance (*C*_*dl*_) can be extracted. The *R*_*s*_ is obtained in the high frequency range of 10^6^ to 10^5^ Hz where the phase is zero, whereas the *R*_*ct*_ associated with the heterogeneous charge transfer at the interface of electrolyte/CE can be estimated from the first semicircle, in the middle frequency range of 10^6^ to 10 Hz. The *R*_*s*_ value refers to the series resistance of the symmetric cell, which includes the sheet resistance of the FTO and contact resistance of the cell. Since EMIMHSO_4_ IL enriched the linkage (alkyl chains) among the PEDOT grains, improved the conductivity due to the anion groups of EMIMHSO_4_, and enhanced the adhesion between PEDOT and FTO substrate, the P-150 CE exhibited better charge transfer kinetics between the FTO substrate and the P-150 layer than bare the PEDOT CE as evidenced by the lower *R*_*s*_ value (20.14 Ω versus 25.12 Ω)^27, 53^. We can also see in Fig. S3 how the ability of adhesion increases. While P-150 well adhered to the surface of FTO, the bare PEDOT tend to easily peeling off from the surface due to its low adherence ability induced by highly polar growth medium. A decrease in *R*_*s*_ can increase the *FF* of a DSSC, yielding photovoltaic performance improvements and leading to the collection of electrons flowing from the external circuit^48^. Furthermore, the *R*_*ct*_ values, which reflect the electrocatalytic activity of the CEs, were 13.61 Ω for bare PEDOT CE and 7.08 Ω for P-150 CE. The P-150 CE, which has the smaller *R*_*ct*_ value, exhibits greater electrocatalytic activity and better charge transfer at the electrolyte/CE compared with the IL-free CE. The decrease in *R*_*ct*_ can be attributed to the increased catalytically active surface area (see Fig. 2) and the enhanced conductivity of the P-150 CE. Owing to the fact that the hydrogen sulphate based ionic liquid employs an important part of polymer matrix with its attractive properties of imidazolium cation and hydrogen sulphate anion in the structure. Besides, the studies in literature show that the alkyl chain length of imidazolium cation and the number of lone pair electrons of anion affect and make an improvement in resonance effectiveness, conductivity, hydrophobicity, intrinsic heterogeneous kinetic parameters for ionic liquid doped-PEDOT films^27, 54, 55^. Furthermore, by lowering the value of *R*_*ct*_, the internal energy loss of the DSSC can be reduced, and the energy conversion efficiency of the cell consequently increased^56^. In addition, a larger *C*_*dl*_ value (10.98 µF) was observed in the P-150 CE than was observed for the bare PEDOT CE (2.67 µF), indicating that a superior reaction area was provided by the P-150 CE due to its larger surface area^57^.

Tafel polarization curves, shown in Fig. 4b, were used to further investigate the electrocatalytic activity and diffusion ability of the electrolyte on surfaces of the P-150 and bare PEDOT CEs. The same cells were used as were employed in the EIS experiments. The Tafel polarization curve theoretically can be divided into three parts: i) the curve forming at low potentials of |V| < 0.12 V defines the polarization region, ii) the curve with a sharp slope at mid-range potentials represents the Tafel zone and iii) the horizontal part of the Tafel plot occurring at high potentials represents the diffusion zone^40^. The intercept of the linear fitting lines of the cathodic and anodic branches in the Tafel zone is defined as the exchange current density (*J*_*0*_). As depicted in Fig. 4b, *J*_*0*_, which is directly associated with electrocatalytic activity, is much higher for the P-150 CE than for the bare PEDOT CE. Thus, ${I}_{3}^{-}$ is reduced to ${I}^{-}$ more effectively with the P150 CE than the bare PEDOT CE. Moreover, the diffusion coefficient (*D*) of triiodide on the surface of the CEs can be calculated using the limiting diffusion current density (*J*_*lim*_), which is extracted from the diffusion zone of the Tafel plot using the following equation:

$$D=\frac{{J}_{\mathrm{lim}}l}{2FnC}$$

where *C* is the concentration of ${I}_{3}^{-}$ (0.1 M), *l* is the spacer thickness (20 µm) between two identical CEs, *F* denotes the Faraday constant, and *n* is the number of electrons involved in the reduction reaction. The *J*_*lim*_ values obtained from the diffusion region for P-150 and bare PEDOT CEs were 1.14 and 0.78 mA/cm^2^, respectively. The *D* value for P-150 CE was 11.82 × 10^−8^ cm^2^/s, higher than that calculated for bare PEDOT CE (8.08 × 10^−8^ cm^2^/s), demonstrating that P-150 CE has a faster diffusion velocity of the ${I}_{3}^{-}/{I}^{-}$ couple in the electrolyte than bare PEDOT CE. The results of the CV, EIS and Tafel-polarization analyses are in agreement and demonstrate that the P-150 CE has greater catalytic activity and better charge transfer kinetics than bare PEDOT CE.

**Figure 5:**
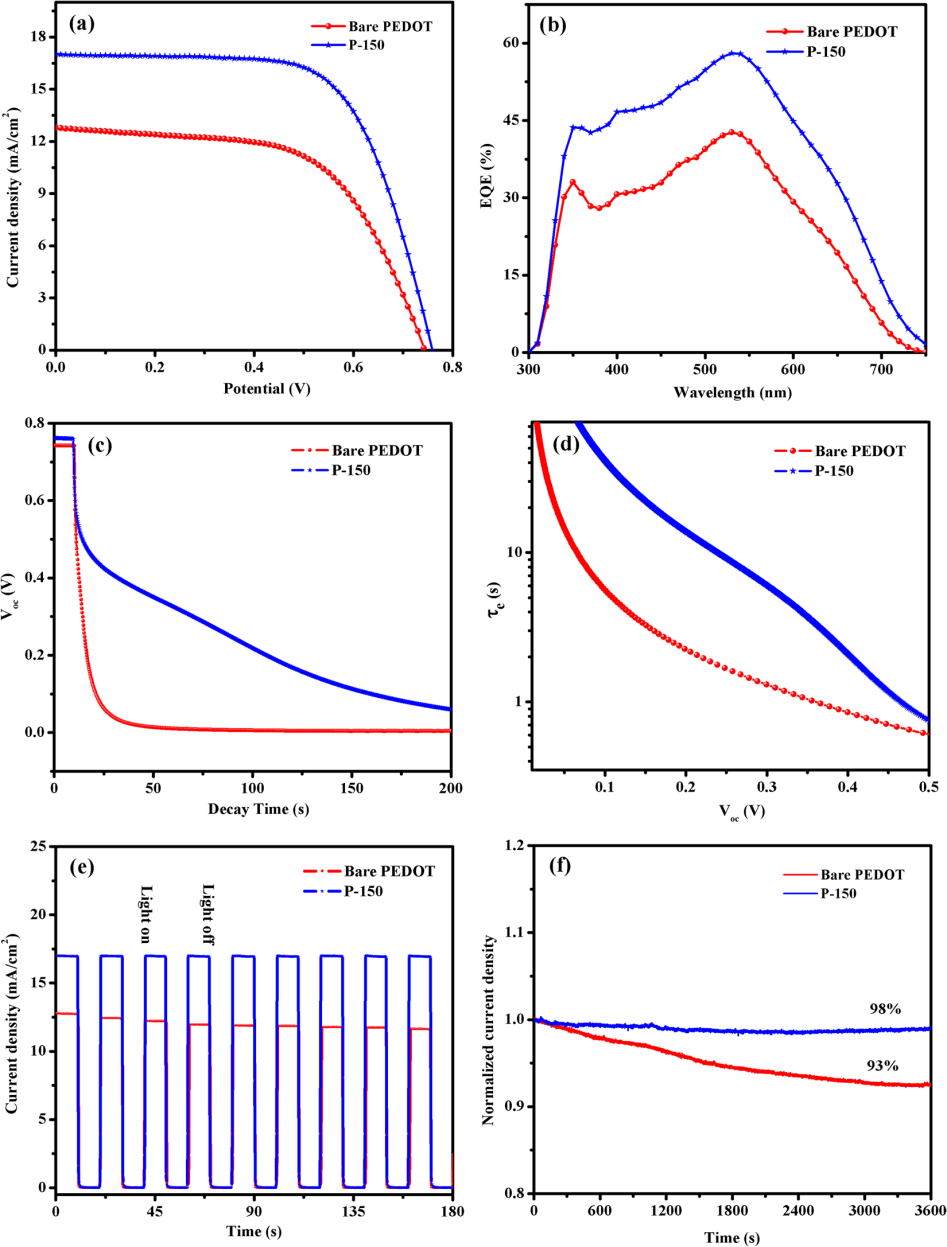
(**a)** Photocurrent density-potential curves of CEs, (**b)** IPCE plots of the DSSCs with P-150 and bare PEDOT CEs, (**c)** OCVD curves (**d)** electron lifetime profiles, (**e)** multiple start/stop cycles and (**f)** photo-current stability of the DSSCs using bare PEDOT and P-150 CEs.

The photocurrent density-potential (J–V) characteristics of the DSSCs with the P-150 and bare PEDOT CEs were recorded under irradiation of 100 mW cm^−2^ (Fig. 5a). The DSSC with a P-150 CE exhibited photovoltaic parameters of an open-circuit voltage (*V*_*oc*_) = 0.76 V, *J*_*sc*_ = 16.99 mA cm^−2^, *FF* = 66.0% and PCE (*η*) = 8.52%. In contrast, the DSSC based on the bare PEDOT CE demonstrated lower photovoltaic parameters (*V*_*oc*_ = 0.74 V, *J*_*sc*_ = 12.79 mA cm^−2^, *FF* = 59.5% and *η* = 5.63%). The inferior PCE value of the cell with bare PEDOT CE can be attributed to the lower *J*_*sc*_ and *V*_*oc*_ values that result from the high *R*_*ct*_ and low electrocatalytic activity through the triiodide reduction (lower *J*_*pc*_ and *J*_*0*_ values) as well as the low *FF* that is due to its much higher *R*_*s*_ value and resulting internal energy loss^58^. Similar IPCE spectra were observed for the DSSCs based on bare PEDOT and P-150 CEs and are shown in Fig. 5b. In the spectral range of 450–600 nm, the IPCE spectra of the DSSCs using bare PEDOT and P-150 CEs were approximately 35 and 50%, respectively. At 550 nm, the IPCE spectra reached maximum values of 43 and 58% for the DSSCs based on bare PEDOT and P-150 CEs, respectively. Furthermore, the IPCE curve obtained using bare PEDOT and P-150 CEs resulted in well-matched photocurrent *J*_*sc*_ values, suggesting that the response of the cells is linear with illumination intensity. Moreover, OCVD analysis was employed to assess the electron recombination processes in the bare PEDOT and P-150 CEs based DSSCs. The OCVD analysis is performed by switching off the illumination in a steady state and monitoring the subsequent decay of photo-voltage. By OCVD measurement useful information about the electron recombination processes and electron life time (${\tau }_{e}$) can be provided. Figure 5c exhibits OCVD curves of the DSSCs based on bare PEDOT and P-150 CEs. Furthermore, ${\tau }_{e}$ for these DSSCs under open-circuit condition is determined by using following equation;

$${\tau }_{e}=\frac{{k}_{B}T}{q}{(\frac{d{V}_{oc}}{dt})}^{-1}$$

where *k*_*B*_ *T* is the thermal energy and *q* is the elementary charge. From the Fig. 5d, we can see that the lifetime depends strongly on Voc. We can divided two voltage dependent regions in which the lifetime is dominated by different factors: (1) the constant lifetime at high photovoltage (>0.5 V), related to both free electrons and internal trapping/detrapping in TiO_2_/dye interface, means that does not depend on photoanode; (2) exhibiting the longer lifetime and slower recombination rate of P-150 in comparison to bare PEDOT CE at low photovoltage (0.5 V<), that corresponds to the reciprocal of the density of levels of acceptor electrolyte species, including the Marcus region^59, 60^. We suspect that this difference at low photovoltage is related to higher catalytic activity of P150 than bare only at the CE in regenerating the oxidized electrolyte which in turn reduces the recombination processes^61^, and enhancing the adherence of PEDOT on FTO, leading to faster electron transfer kinetics at the CE/electrolyte interface and lower electron recombination rate at the substrate/electrolyte interface^62^, but further work is required to clarify this effect.

In order to explore the charge transfer and charge-recombination processes of the IL-assisted and IL-free PEDOT, the photoresponse measurements were performed in the DSSC at 0 V under illumination. Figure 5e displays the transient photocurrent response for bare PEDOT and P-150 CEs. It is noted that there is a fast and uniform photocurrent responding to each switch-on/off event in the IL-assisted counter electrode. Moreover, the photocurrent is so stable that no obvious photocurrent decay is observed after eight start/stop cycles. In contrast, the electrodes with IL-free bare electrode exhibit low photocurrent density and decay 8% from initial value. The improved photocurrent performance with hydrogen sulphate-based IL should be ascribed to the following factors: i) the strong adhesion of PEDOT on FTO surfaces, ii) enhanced electrical conductivity, iii) forming porous structure, and iv) superior electrocatalytic activity for reduction of ${I}_{3}^{-}$ species^63, 64^. To examine the stability of the bare PEDOT and P-150 CEs under prolonged irradiation, the *J*_*sc*_ of the assembled DSSC was also recorded with irradiation over 3600 s. As shown in Fig. 5f, ~98% and ~93% of the initial normalized *J*_*sc*_ remained in the cells with P-150 and bare PEDOT CEs, respectively. This result suggests relatively good stability of the solar cell with a P-150 CE in comparison to bare PEDOT. Although the DSSC tested for only 3600 s, this preliminary result demonstrates that the stability of a DSSC can be prolonged by employing a P-150 CE.

### Part B: Effect of polymerization charge capacity on the EMIMHSO_4_ IL-doped PEDOT CEs

**Figure 6:**
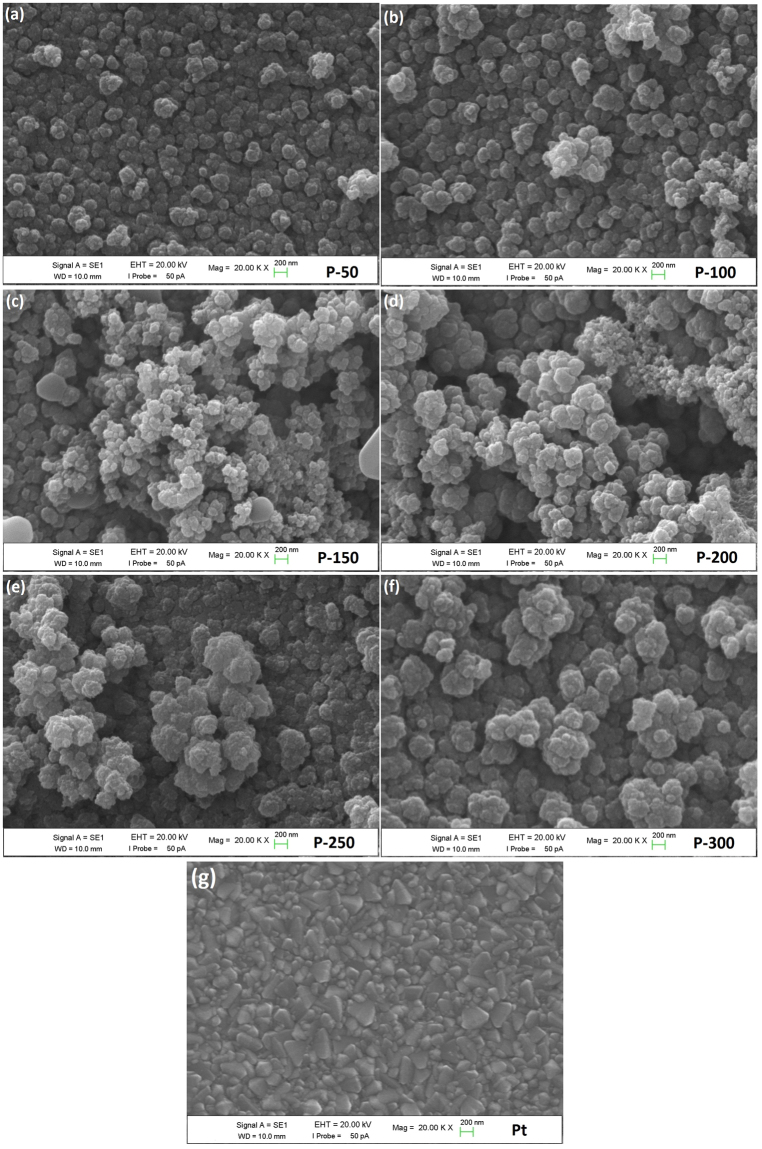
SEM micrographs of (**a**–**f)** electropolymerized PEDOT CEs at various deposition charges and (**g)** Pt CE.

The influence of the deposition charge capacity on the conformation of the electro-polymerized PEDOT with IL was also investigated using the SEM micrographs, as shown in Fig. 6a–f. The Pt CE shows a smooth surface morphology with a low degree of porosity (Fig. 6g), whereas the electro-polymerized PEDOT CEs exhibit highly porous morphologies (Fig. 6a–e). Furthermore, as exhibited in Fig. 6a–f, as the electro-polymerization charge capacity increased from 50 to 150 mC/cm^2^, the surface changed from dense to more porous with small globular grains. Further increase in the charge capacity from 200 to 300 mC/cm^2^ led to significant aggregations and increase in the grain size that reduced the catalytically active surface area due to the increase in the film thickness. The increase in film thickness is ascribed to the increased polymerization rate with the increase of the polymerization charge capacity. The decrease in the active surface area can result in low electrocatalytic activity and *J*_*sc*_ values of the DSSC applications^65^. Therefore, the electro-polymerization charge capacity of 150 mC/cm^2^ is most suitable for CE applications.

**Figure 7:**
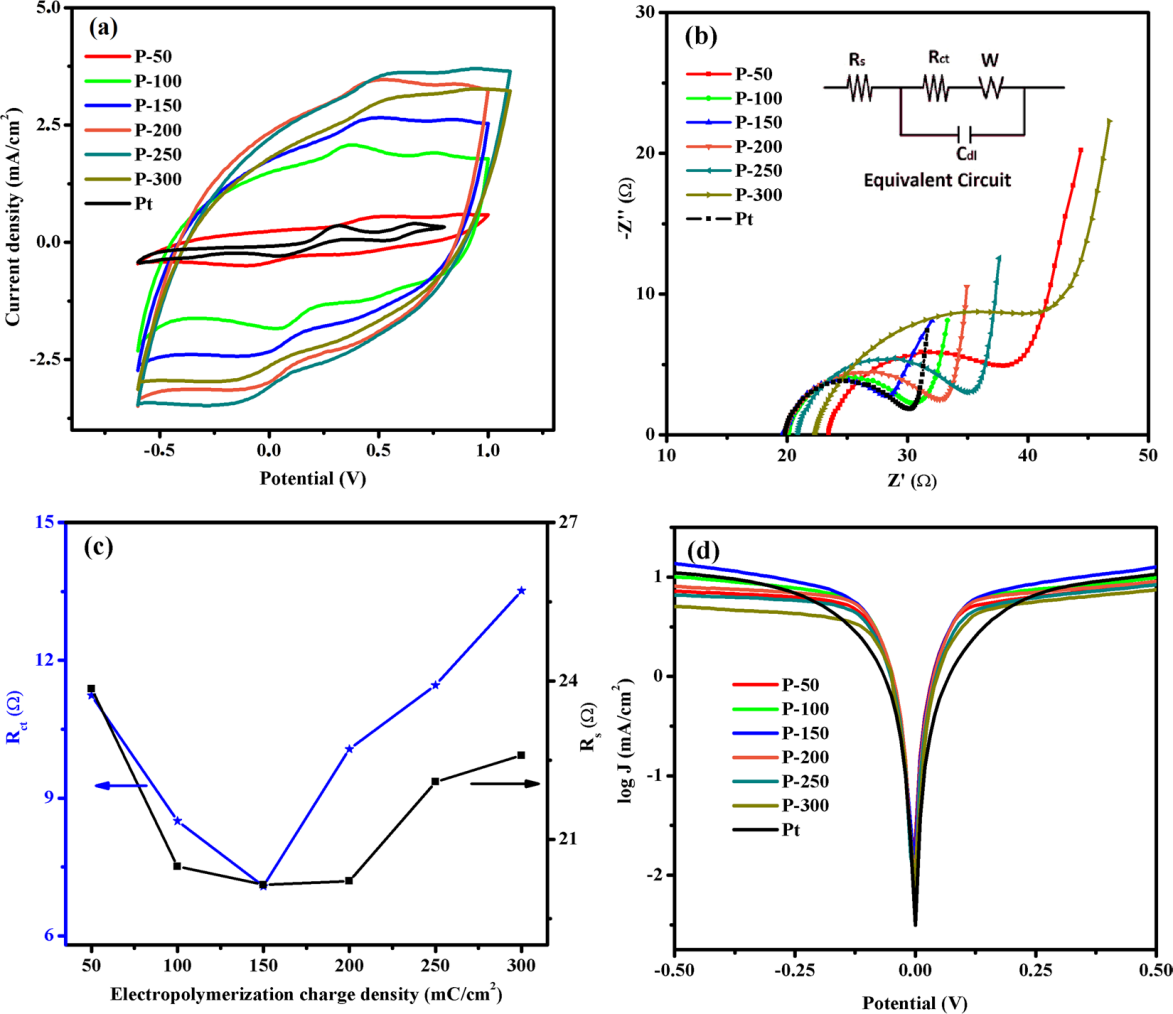
(**a)** CV plot, (**b)** Nyquist plot and equivalent circuit (inset), (**c)** *R*_*ct*_ and *R*_*s*_ versus electro-polymerization charge density and (**d)** Tafel polarizations of dummy cells with prepared CEs of P-50, P-100, P-150, P-200, P-250 and P-300 as well as Pt.

The CV studies of the P-50, P-100, P-150, P-200, P-250, P-300 and Pt CEs at a scan rate of 100 mVs^−1^ are shown in Fig. 7a. Well defined redox peaks were observed for all of the CEs due to the high level reduction of triiodide species in the electrolyte. As clearly shown in Fig. 7a, all the PEDOT CEs demonstrated much higher *J*_*pd*_ values as compared to Pt CE. This trend can be ascribed to the large catalytically active site of PEDOT CEs for storing the electrolyte, implying a better electrocatalytic activity for the reduction of triiodide ions^65, 66^. Moreover, the *J*_*pd*_ values increased as the polymerization charge capacity increased up to 250 mC/cm^2^; at 300 mC/cm^2^, the *J*_*pd*_ value decreased due to resulting changes in the porosity level and the film thickness of PEDOT CEs^67^. However, the *E*_*pp*_ value increased as the polymerization charge capacity increased because the oxidation peak shifted slightly toward the negative potential, suggesting that the electrocatalytic activity decreases as the polymerization charge capacity increases. This increase in *E*_*pp*_ can result in overpotential loss of the corresponding DSSCs^68^. Taken together, the *J*_*pd*_ and *E*_*pp*_ parameters of the P-50, P-100, P-150, P-200, P-250 and P-300 CEs indicate that the optimum electro-polymerization charge capacity is 150 mC/cm^2^ for efficient reduction reaction of triiodide in the electrolyte.

To understand the impact of electro-polymerization charge capacity on the interfacial charge-transfer ability of the CEs, EIS measurements were made, and the resulting Nyquist curves are displayed in Fig. 7b. The *R*_*s*_, *R*_*ct*_ and *C*_*dl*_ parameters, dictating the electrocatalytic activity of CE materials, extracted from the equivalent circuit are listed in Table 1. As shown in Fig. 7c, the *R*_*ct*_ reduced from 11.24 to 7.08 Ω for deposition charge capacities of 50–150 mC/cm^2^ and further increased when the charge capacity exceeded 200 mC/cm^2^. This can be attributed to the doping level of the functional group in the EMIMHSO_4_ IL. The doping level in PEDOT CEs may decrease as the polymerization rate increases in response to the electro-polymerized charge capacity (200 to 300 mC/cm^2^); thus, the sheet resistance can increase, and the conductivity can decline^67^. Moreover, the increase of *R*_*ct*_ also can be ascribed to the decrement in active surface area and porosity of CEs (see the Fig. 6e,f). Because of its large catalytically active surface area and good catalytic property for the reduction of triiodide, a charge capacity of 150 mC/cm^2^ was associated with the smallest *R*_*ct*_ value. Similar behavior is observed for the *R*_*s*_ values in the literature and can be attributed to the tendency of thick films to peel off from the surface of the substrate or the decrease in the conductivity, which results from the increase of polymerization charge capacity^40, 41^. Furthermore, P-150 CE showed lower *R*_*s*_ and *R*_*ct*_ values compared with Pt CE, indicating that P-150 CE possesses excellent electrocatalytic activity through the reduction of ${I}_{3}^{-}$ to ${I}^{-}$ and superb charge transfer kinetics. Furthermore, the P-150 CE demonstrated the highest *C*_*dl*_ value, proving that the P-150 CE has more active surface area compared with the Pt CE and the other PEDOT CEs. Tafel polarization measurements were also conducted to confirm the electrocatalytic activity of the fabricated CEs. The slopes in the Tafel region were compared to determine the *J*_*0*_ on the CE surface, which changes inversely with *R*_*ct*_. As shown in Fig. 7d, as expected, from 50–150 mC/cm^2^, *J*_*0*_ increases as the polymerization charge capacity increases; however, further increase in charge capacity from 200 to 300 mC/cm^2^ results in lower *J*_*0*_ values. Moreover, the determined *D* values from the Tafel plots in the diffusion zone reveal that the P-150 CE exhibits a faster diffusion velocity of the ${I}_{3}^{-}/{I}^{-}$ couple in the electrolyte than Pt CE. Thus, the SEM, CV, EIS and Tafel polarization analyses are in good agreement.

**Table 1:** The obtained electrochemical and photovoltaic parameters of P-50, P-100, P-150, P-200, P-250, P-300 and Pt CEs.

Samples	*R*_*s*_ (Ω)	*R*_*ct*_ (Ω)	*C*_*µ*_ (µF)	*J*_*lim*_ (mA/cm^2^)	*D* × 10^−8^ (cm^2^/s)	*V*_*oc*_ *(V)*	*J*_*sc*_ *(mA/cm* ^2^)	*FF (%)*	${\boldsymbol{\eta }}\,({\boldsymbol{ \% }})$
P-50	23.85	11.24	9.75	0.86	8.91	0.75	13.35	64.1	6.42
P-100	20.49	8.50	10.55	1.00	10.36	0.76	14.99	65.1	7.42
P-150	20.14	7.08	10.98	1.14	11.82	0.76	16.99	66.0	8.52
P-200	20.21	10.07	10.74	0.91	9.43	0.75	15.49	63.1	7.33
P-250	22.09	11.46	10.09	0.82	8.50	0.74	11.70	62.0	5.37
P-300	22.59	13.52	10.29	0.71	7.36	0.74	10.53	62.0	4.83
pt	20.21	8.33	7.85	1.04	10.78	0.77	15.87	64.4	7.87

**Figure 8:**
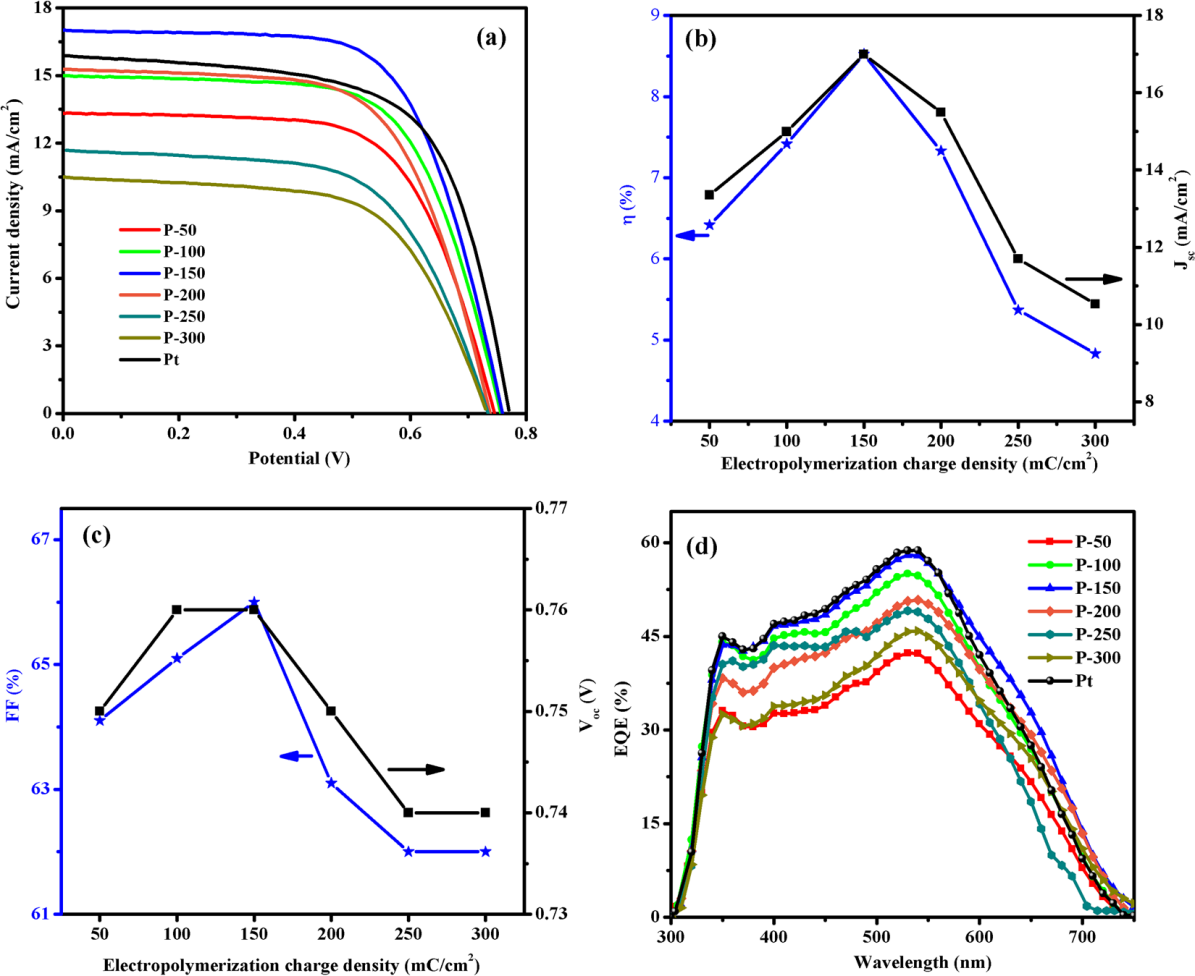
(**a)** Photocurrent density-potential curves, (**b)** plots of *η* and *J*_*sc*_, (**c)** *FF* and *V*_*oc*_ versus electro-polymerization charge density and (**d)** IPCE plots of the DSSCs equipped with P-50, P-100, P-150, P-200, P-250, P-300 and Pt CEs.

The *J-V* characteristics of the DSSCs with various CEs, including P-50, P-100, P-150, P-200, P-250, P-300 and Pt films, were obtained at 100 mW/cm^2^ and are shown in Fig. 8a. The photovoltaic parameters with various electro-polymerization charge capacities of PEDOT CE are summarized in Fig. 8b,c and Table 1. As depicted in Fig. 8b,c, upon increasing the PEDOT film thickness by increasing the polymerization charge density from 50 to 150 mC/cm^2^, *J*_*sc*_ value improved from 13.35 to 16.99 mA/cm^2^, which is higher than the *J*_*sc*_ obtained with the Pt CE (15.87 mA/cm^2^). This improvement in *J*_*sc*_ can be attributed to a large surface area and excellent electrocatalytic activity, which were confirmed by SEM, CV, EIS and Tafel polarization analyses for the P-150 CE^50^. Furthermore, the *J*_*sc*_ value decreased from 15.49 to 10.53 mA/cm^2^ as the electro-polymerization charge capacity increased from 200 to 300 mC/cm^2^ due to the significant aggregation of PEDOT polymer. Reports of a similar trend observed for *J*_*sc*_ values in response to changing the thickness of the CE are noted in the literature^69, 70^. The obtained *FF* values of the fabricated DSSCs exhibited similar behavior with *J*_*sc*_ in response to changes in electro-polymerization charge capacity. However, the *V*_*oc*_ value was noted as 0.75 V for P-50 CE, 0.76 V for P-100 and P-150 CEs, 0.75 for P-200, 0.74 for P-250 and P-300 CEs, and 0.77 V for Pt CE. Based on the CV analyses, the opposite trend was observed for the *E*_*pp*_ values, which leads to overpotential loss at the FTO/CE material interface or the electrolyte/CE material interface^41^. Optimal photovoltaic parameters such as *J*_*sc*_, *FF* and *V*_*oc*_ can be achieved at 150 mC/cm^2^ electro-polymerization charge density. Moreover, the DSSC with the P-150 CE had the highest *J*_*sc*_ and *FF* and resulted in a high PCE of 8.52% compared with 7.87% for the DSSC with the Pt CE. To further evaluate the photovoltaic performance of the DSSCs based on the P-50, P-100, P-150, P-200, P-250, P-300 and Pt CEs, IPCE spectra of the DSSCs were collected. As depicted in Fig. 8d, the IPCE spectra of the cells were maximized at approximately 550 nm, a finding that agrees well with the photovoltaic parameters obtained from J-V characteristics^66^. The DSSCs with P-150 and Pt CEs exhibited the same IPCE value of 58% in the range of 300–600 nm. However, between 600–700 nm, the DSSC with the P-150 CE showed a slightly higher IPCE value than the cell with the Pt CE, indicating that the P-150 CE possesses enhanced photovoltaic performance compared with the Pt CE.

## Conclusions

In summary, PEDOT CPs have been successfully synthesized by embedding EMIMHSO_4_ ILs with electro-polymerization process to fabricate high efficiency and low-cost Pt-free DSSCs for the first time. Furthermore, the electro-polymerization of EMIMHSO_4_-doped PEDOT was performed at various charge capacities ranging from 50 to 300 mC/cm^2^ in 50 mC/cm^2^ increments. To confirm the impacts of EMIMHSO_4_ and polymerization charge capacity on the performance of the PEDOT CEs, SEM, CV, EIS, Tafel polarization and photovoltaic measurements were conducted. In response to the use of EMIMHSO_4_ IL as supporting media, the surface morphology of the PEDOT changed from a randomly distributed cluster structure to nanometer-sized uniform globular grains. This change in surface morphology resulted in a highly porous surface structure, indicating superb electrocatalytic activity through the reduction of ${I}_{3}^{-}$ to ${I}^{-}$. Furthermore, EMIMHSO_4_ not only changed the surface morphology of the PEDOT films but also improved the adhesion of PEDOT on FTO substrate and film conductivity, which led to excellent interfacial charge transfer kinetics between PEDOT/FTO and PEDOT/electrolyte interfaces. The *R*_*ct*_ and *R*_*s*_ values were obtained as 25.12 and 13.61 Ω, respectively for the bare PEDOT CE, whereas these values were extracted as 20.14 and 7.08 Ω for the P-150 CE. The DSSCs based on bare PEDOT and P-150 CEs demonstrated PCEs of 5.63 and 8.52%, respectively. The photoresponse results confirm that the IL-assisted PEDOT electrode shows the highest photocurrent, extremely efficient charge transport properties, and superior stability of the material compared to bare PEDOT electrode. Furthermore, the optimal charge capacity was 150 mC/cm^2^ due to the larger active surface area, lower *R*_*ct*_ and *R*_*s*_ values, and higher *J*_*sc*_ and *FF* values. More importantly, the DSSC with the P-150 CE produced an excellent PCE of 8.52%, which is higher than that of the Pt-based reference cell (7.87%); the high PCE is mainly attributed to the larger active surface area, higher electrocatalytic activity and better interfacial charge transfer kinetics. The P-150 CE is a promising alternative to the commonly used but expensive Pt CE. Thus, the EMIMHSO_4_-doped PEDOT electro-polymerized at 150 mC/cm^2^ charge capacity offers several attractive advantages such as high catalytic stability, low-cost fabrication, large-scale production potential and high PCE. We hope this proof of concept by directly electro-polymerizing PEDOT doped with EMIMHSO_4_ IL on substrates to provide a serious impression on the development of the green industry such as supercapacitors, water splitting, as well as perovskite solar cells.

### Experimental Section

#### Preparation of PEDOT-based CEs

All electro-polymerization processes were performed employing a three electrode Ivium compactStat potentiostat/galvanostat and chronoamperometry technique. An FTO (Asahi Glass, fluorine-doped SnO_2_; sheet resistance: 15 sq^−1^) and platinum sheet were used as the working and counter electrodes, respectively. Ag/AgCl in 3 M NaCl (aq.) solution was used as the reference electrode. The FTO substrate (1.5 cm^2^) was first cleaned as described previously^71^. Bare PEDOT CE was synthesized in a medium in acetonitrile (ACN) containing 0.01 M EDOT monomer and 0.1 M LiClO_4_. The IL-doped PEDOT counter electrode, P-150, was synthesized in a medium containing 0.01 M EDOT monomer, 0.1 M LiClO_4_ and 0.1 M of EMIMHSO_4_ IL in ACN. The polymer films were obtained via constant potential electrolysis of 1.5 V, and their electrochemical behaviors were investigated in monomer free electrolyte solutions. For electrochemical studies, PEDOT films were obtained on FTO glass in different thickness by varying the polymerization charge from 50 to 300 mC/cm^2^ in 50-mC/cm^2^ increments. After the deposition of PEDOT films, the substrates were washed in ACN solution and annealed at 60 °C for 30 min. The polymer films were switched between neutral and doped states several times to equilibrate the redox behavior in monomer-free electrolytic solution (Fig. 1a). The polymerized PEDOT CEs with IL obtained at various charge capacities (50, 100, 150, 200, 250 and 300 mC/cm^2^) were marked as P-50, P-100, P-150, P-200, P-250 and P-300. In addition, the PEDOT polymerized at 150 mC/cm^2^ without IL was labeled as bare PEDOT. On the other hand, Pt-based CE (as a control electrode) was prepared according to our previous report^9^.

#### Preparation of TiO_2_ photoanodes and redox electrolyte

In the present study, titanium (IV) oxide nanopowders (718467, Sigma Aldrich) based electrodes were sensitized with 0.5 mM of di-tetrabutylammoniumcis-bis(isothiocyanato)bis(2,2’-bipyridyl-4,4’dicarboxylato)ruthenium(II) (N-719) dye (703214, Sigma Aldrich) during the 18 h. The thicknesses of the photoelectrode layers have been optimized as ~12 μm. Therefore, the effect of thickness on photovoltaic performance can be ignored. The process of preparing working electrodes (photoanodes) was detailed in our previous reports^4, 9^. Moreover, as described in previously^71^, to prepare the ${I}_{3}^{-}/{I}^{-}$ redox electrolyte, 0.01 M iodine, 0.6 M 1–butyl–3–methylimidazolium iodide, 0.1 M 4–tert–butylpridine and 0.1 M lithium iodide hydrate were prepared in 3–methoxypropionitrile solvent, separately. Then, the prepared solutions were mixed and subjected to magnetic stirrer for 2 h.

#### The assembly of DSSCs

Assembly of DSSCs was performed as described previously^72^. Briefly, the photoanode was positioned face up on a horizontal surface, and a CE was placed on top of the photoanode. These two opposing electrodes were offset from one another such that the CE covered the entire working electrode. The redox electrolyte was poured at the edges of the electrodes, and the electrolyte was drawn into the space between the two electrodes via capillary action.

#### Characterizations

Fourier transform infrared (FTIR) spectroscopy of the EDOT, EMIMHSO_4_ ionic liquid, EMIMHSO_4_ doped PEDOT and bare PEDOT CEs electro-polymerized at 150 mC/cm^2^ charge capacity was carried out between 4000 and 400 cm^−1^ range using a Jasco FTIR-430 spectrometer. To examine surface morphologies of the CEs, scanning electron microscopy (SEM) was carried out using Zeiss Evo model SEM. Furthermore, the surface morphologies of bare PEDOT and P-150 CEs were investigated using Nanomagnetic Instrument model of atomic force microscopy (AFM) at tapping mode. In addition, the hydrophilicity of the bare PEDOT and P-150 film surfaces was assessed by the contact angle measurement by use of Attension Theta Lite model contact angle meter system. The current density-potential (*J-V*) curves of fabricated DSSCs were obtained using Keithley 4200 semiconductor characterization system using OAI Class AAA solar simulator under the AM 1.5 G illumination of 100 mW cm^−2^ as the light source. Incident photon to current efficiency (IPCE) of the cells was investigated using an Enlitech QE-R system with a 75 W xenon arc lamp source. Furthermore, the open circuit voltage decay (OCVD), multiple start/stop (on−off) cycles and photo-current density (*J*_*sc*_) stability profiles of the DSSCs assembled with bare PEDOT and P-150 CEs were recorded by a set up including OAI Class AAA solar simulator and Ivium compactStat system. Firstly, at the very beginning of the OCVD measurement, the DSSCs were illuminated by the solar simulator for a steady voltage, and then the light was switched off and OCVD profiles were recorded using chronopotentiometry mode of the Ivium compactStat system. Secondly, the multiple start/stop switches were obtained using chronoamperometry mode of the Ivium compactStat system by alternating the irradiation of the solar simulator with intensities of 100 and 0 mW cm^−2^. Thirdly, *J*_*sc*_ stability profiles of the cells were recorded using chronoamperometry mode of the Ivium compactStat system under sustained irradiation of 100 mWcm^−2^ for 3600 s. The cyclic voltammetry (CV) and 50 consecutive voltammetry cycles of CEs was carried out in electrolytic medium containing 0.1 M LiClO_4_, 0.1 M ${I}_{3}^{-}/{I}^{-}$ redox electrolyte and ACN at scan rate of 100 mV s^−1^ and in range of −0.6–1.0 V, using three electrode set up same as used for electro-polymerization process. For electrochemical impedance spectroscopy (EIS) and Tafel polarization measurements, the symmetrical dummy cells were assembled with two identical CEs filled with the same redox electrolyte as used in the fabrication of DSSCs. The active area of the dummy cells was 0.64 cm^2^. In EIS tests, the samples were scanned from 100 Hz to 100 kHz at 0.70 V forward bias with 10 mV AC amplitude. The polarization measurements were performed in range of −0.5 and 0.5 V at a scan rate of 10 mV s^−1^. All the electrochemical analyses were performed using an Ivium compactStat system.

## Electronic supplementary material

SUPPLEMENTARY INFO
