# Supplementary material for: Hydrogen sulphate-based ionic liquid-assisted electro-polymerization of PEDOT catalyst material for high-efficiency photoelectrochemical solar cells
Source: Sci Rep. 2017 Sep 15;7:11672. doi: 10.1038/s41598-017-11916-4 (PMC5600988; doi:10.1038/s41598-017-11916-4)
Supplement: Supplementary file 1 — SUPPLEMENTARY INFO [file 41598_2017_11916_MOESM1_ESM.doc]

**Supporting Information**

for

**Hydrogen sulphate-based ionic liquid-assisted electro-polymerization of PEDOT catalyst material for high-efficiency photoelectrochemical solar cells**

by

*Buket Bezgin Carbas,^1,2^, Mahir Gulen^3,4^, Merve Celik Tolu^1^, Savas Sonmezoglu^3,4*^*

*^1^Department of Energy Systems Engineering, Karamanoglu Mehmetbey University, Karaman, Turkey*

*^2^Conductive Polymers and Energy Applications Laboratory, Karamanoglu Mehmetbey University, Karaman, Turkey*

*^3^Nanotechnology R&D Laboratory, Karamanoglu Mehmetbey University, Karaman, Turkey*

*^4^Department of Metallurgical and Materials Engineering, Karamanoglu Mehmetbey University, Karaman, Turkey*

**
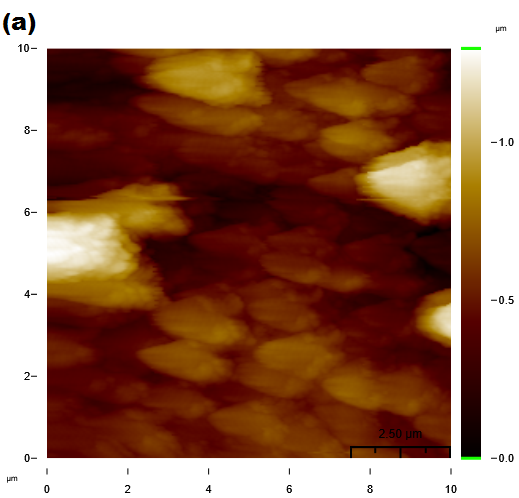

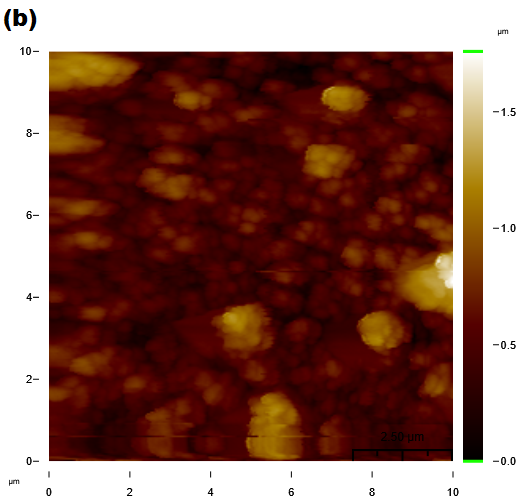
**

**Figure S1.** AFM image of **a**) bare PEDOT and **b)** P-150 CEs.

**Figure S2.** Nyquist plots of bare PEDOT and P-150 electrodes in the medium of 0.1 M LiClO_4_/ACN.

**
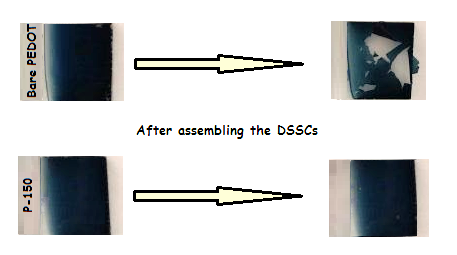
**

**Figure S3.** Picture of bare PEDOT and P-150 CEs after assembling of the DSSCs.

**Table S1. ATR band positions and associating modes for EDOT monomer, EMIMHSO_4_, bare PEDOT and P-150 films.**

| **Wavenumbers (cm^-1^)** | | | **Band Assignments** | **Ref.** |
| --- | --- | --- | --- | --- |
| **Bare PEDOT** | **EMIMHSO_4_** | **P-150** |
| - | 3150, 3105 | 3150, 3105 | C-H stretching of imidazole ring | [1,2] |
| 2929, 2861 | 2982 | 2931, 2851 | CH_2_ stretching | [2] |
| 1521, 1631 | 1576 | 1541, 1675 | Stretching modes of C=C and C-C in the thiophene ring, C=N stretching | [1,3] |
| 1389, 1336 | 1466 | 1411, 1336 | CH_2_ asymmetric, symmetric wagging | [1,2] |
| - | 1220 | 1220 | CH_2_ asymmetric twisting, -O-SO_3_ asymmetric switching | [1,2] |
| 1190, 1142 | 1166 | 1146 | C–O stretching in the ethylenedioxyl group, -N-CH2, -N-CH_3_ stretching, S-O vibrations of SO_4_ | [1,3,4] |
| 1090 | 1026 | 1090 | C-O-C symmetric assymetric stretching, O-SO_3_ symmetric stretching, dopant ClO_4_^-^ | [1,5] |
| 980, 921,  840 | 836, 752 | 980, 921, 836, 756 | C–S vibration of the thiophene, S-OH stretching, CH_2_ rocking | [6] |

**Abbreviations of Ionic Liquids**

**EMImTf_2_N**: 1-ethyl-3-methylimidazolium bis(trifluoromethylsulfonyl)amide [7].

**LiTf_2_N**: Lithium bis(trifluoromethanesulfone)imide [7].

**BMITFSI**: 1-Butyl-3-methylimidazolium bis(trifluoromethylsulfonyl)imide [8].

**BMPyTFSI**: 1-butyl-3-methylpyridnium bis(trifluoromethylsulfonyl)imide [8].

**EMIFAP**: 1-ethyl-3-methylimidazolium tris(pentafluoroethyl) trifluorophosphate [8].

**EMIBF_4_**: 1-ethyl-3-methylimidazolium tetrafluoroborate [9].

**HMIBF_4_**: 1-hexyl-3-methylimidazolium tetrafluoroborate [9].

**DMIBF_4_**: 1-dectyl-3-methylimidazolium tetrafluoroborate [9].

**HMIPF_6_**: 1-hexyl-3-methylimidazolium hexafluorophosphate [9].

**HMISO_3_CF_3_**: 1-hexyl-3-methylimidazolium trifluoromethansulfonate [9].

**HMITFSI**: 1-hexyl-3-methylimidazolium bis(trifluoromethylsulfonyl)imide [9].

**References**

[1] J. Kiefer, J. Fries and A. Leipertz, *Appl. Spectrosc*., 2007, **61**, 1306–1311.

[2] N.R. Dhumal, H.J. Kim and J. Kiefer, *J. Phys. Chem. A,* 2011, **115**, 3551–3558.

[3] Q. Che, R. He, J. Yang, L. Feng and R.F. Savinell, *Electrochem. Commun.,* 2010, **12**, 647–649.

[4] D.T. Chin and H.H. Chang, *J. Appl. Electrochem*., 1989, **19**, 95–99.

[5]A. Cihanerand, F.Algı, *Electrochim. Acta*, 2008, **54**, 665–670.

[6] M. Gargouri, T. Mhiri, A. Daoudand J.M. Réau, *Solid StateIonics*, 1999, **125**, 193–202.

[7] R. Senadeera, N. Fukuri, Y. Saito, T. Kitamura, Y. Wada and S. Yanagida, *Chem. Commun.,* 2005, **7**, 2259–2261.

[8] S. Ahmad, J.H. Yum, Z. Xianxi, M. Graetzel, H.J. Butt and M.K. Nazeeruddin, *J. Mater. Chem.,* 2010, **20**, 1654–1658.

[9] C.T. Li, C.P. Lee, M.S. Fan, P.Y. Chen, R. Vittal and K.C. Ho, *Nano Energy*, 2014, **9**, 1–14.
